# Total neoadjuvant therapy for locally advanced rectal cancer: a three-group propensity score matched study

**DOI:** 10.1007/s00384-024-04610-1

**Published:** 2024-03-16

**Authors:** Jiahao Zhou, Jun Huang, Zikai Zhou, Xiangbing Deng, Qingbin Wu, Ziqiang Wang

**Affiliations:** 1 Department of General Surgery, Colorectal Cancer Center, West China Hospital, Sichuan University, No. 37, Guo Xue Xiang, Chengdu, 610041 China; 2 West China School of Medicine, Sichuan University, Chengdu, 610041 China

**Keywords:** Rectal cancer, Total neoadjuvant therapy, Chemoradiotherapy, Disease-free survival, Metastasis-free survival

## Abstract

**Purpose:**

Total neoadjuvant therapy (TNT) has emerged as a therapeutic approach for locally advanced rectal cancer (LARC). However, the optimal chemotherapy cycles within TNT remain uncertain. This study aimed to evaluate and compare the prognostic efficacy of varying cycles of chemotherapy during TNT for LARC.

**Methods:**

Patients diagnosed with LARC (T3-4N0M0/T1-4N1-2M0), who underwent TNT or chemoradiotherapy followed by total mesorectal excision (TME) between 2015 and 2020, were retrospective included. Patients were categorized into three groups based on their neoadjuvant strategy: CRT (long-course chemoradiotherapy), STNT (long-course CRT with one to three cycles of chemotherapy), and LTNT (long-course CRT with four or more cycles of chemotherapy). Propensity score matching (PSM) based on gender, age, body mass index, tumor distance from the anal verge, clinical T stage, clinical N stage, and mesorectal fascia status was employed to reduce confounding bias. Primary endpoints were disease-free survival (DFS) and metastasis-free survival (MFS).

**Results:**

The study comprised 372 patients, with 73 patients in each group after PSM. Compared with CRT, both STNT and LTNT demonstrated improved DFS (5-year rate: 59.7% vs. 77.8% vs. 76.5%, p = 0.027) and MFS (5-year rate: 65.1% vs. 81.3% vs. 81.4%, p = 0.030). There was no difference in DFS or MFS between STNT and LTNT. These favorable outcomes were consistent among subgroups defined by tumor distance from the anal verge ≥ 5 cm, clinical T3 stage, clinical N positive status, or involved mesorectal fascia.

**Conclusion:**

Compared to CRT, both STNT and LTNT demonstrated improved DFS and MFS outcomes. Notably, survival outcomes were similar between STNT and LTNT, suggesting that chemotherapy cycles in TNT may not significantly impact survival.

**Supplementary Information:**

The online version contains supplementary material available at 10.1007/s00384-024-04610-1.

## Introduction

Neoadjuvant chemoradiotherapy (CRT) followed by total mesorectal excision (TME) and adjuvant chemotherapy has been the standard treatment for locally advanced rectal cancer (LARC) during a long period due to the improvement of local control [1, 2]. However, approximately one-third of patients still experience distant metastases [3–5], often attributed to delayed or inadequate compliance to adjuvant chemotherapy regimens. Consequently, total neoadjuvant therapy (TNT), which delivers chemotherapy before CRT (induction chemotherapy) and/or after CRT (consolidation chemotherapy) prior to surgery, has gained traction. Various trials have compared TNT with CRT. For instance, the phase III PRODIGE-23 trial found that TNT significantly improved compliance to chemotherapy, pathological complete response (pCR) rate, and disease-free survival (DFS) compared with CRT [6]. Another large cohort study form the Memorial Sloan Kettering Cancer Center (MSKCC) also showed that TNT improved the compliance to chemotherapy and pCR rate, but failed to improve DFS, as well as our recent meta-analysis [7–9]. A consensus has been reached on enhancing primary tumor downstaging through TNT. However, whether it can improve survival outcomes remains controversial. One reason might be that previous studies employed varying cycles of chemotherapy during TNT [6–8, 10–12], and as a consequence, limited data on studies could be found to compared the efficacies of different cycles of chemotherapy during TNT. Although the National Comprehensive Cancer Network (NCCN) guideline and the European Society of Medical Oncology (ESMO) recommended TNT as a treatment choice for LARC, they did not specify the optimal cycles of chemotherapy either [13, 14]. Herein, the current study aimed to use propensity score matching (PSM) to assess and compare the prognostic efficacy of different cycles of chemotherapy during TNT for LARC.

## Methods

Patients with LARC who underwent TNT or CRT followed by TME surgery at our hospital between January 2015 and December 2020 were retrospectively included. Data were obtained from a prospective colorectal cancer database approved by the ethics committee of our hospital. Patient characteristics, perioperative and pathological outcomes, and survival data were extracted. Computed tomography, high-definition magnetic resonance imaging, and/or endorectal ultrasonography were used for clinical staging in accordance with the American Joint Committee on Cancer TNM staging standard of rectal cancer (8th edition).

This study adhered to the STROBE guidelines.

## Inclusion and exclusion criteria

Patients meeting the following criteria were included: 1) biopsy-confirmed rectal adenocarcinoma; 2) patients with LARC, defined as clinical stage II-III, who received TNT/CRT followed by TME. Exclusion criteria comprised: 1) the distance of tumor from the anal verge measured by colonoscopy > 12 cm; 2) recurrent tumor or distant metastasis; 3) concurrent malignancy in any other organs; 4) neoadjuvant chemotherapy or short-course radiotherapy followed by TME; 5) immunotherapy or target therapy; 6) transanal local excision or watch & wait strategy after TNT/CRT.

## Neoadjuvant treatment and groups

Patients with LARC were advised to receive neoadjuvant therapy according to ESMO guidelines [15] and their preferences. According to the neoadjuvant treatment strategy, patients were categorized into CRT, STNT, and LTNT group. The CRT was defined as patients who received long-course CRT alone (50.4 Gy/25-28f radiation therapy with concurrent capecitabine). The STNT was defined as patients who received long-course CRT with a cumulative total of one to three cycles of induction and/or consolidation CAPOX (oxaliplatin and capecitabine) or FOLFOX (oxaliplatin, leucovorin, and fluorouracil). The LTNT was defined as patients who received long-course CRT with a cumulative total of four or more cycles of induction and/or consolidation CAPOX or FOLFOX.

## Surgery and follow-up

Surgery was performed 8–12 weeks after the end of radiation or 4–6 weeks after chemotherapy. The surgical procedure followed the TME principle proposed by Heald, which was described in our previous work [16, 17]. Adjuvant chemotherapy was performed according to the recommendation of ESMO guidelines [15] and patients’ own willingness. All patients were advised to undergo regularly follow-up examinations, including CEA, CA199, and chest and abdominopelvic computed tomography scans every six months during the first three years after surgery and annually thereafter. The detailed follow-up schedule was described previously [17]. The last follow-up was completed in January 2023.

## End points

The primary endpoints were disease-free survival (DFS) and metastasis-free survival (MFS). DFS was defined as the time from the beginning of neoadjuvant treatment to the first occurrence of distant metastasis, local recurrence, or death from any cause. MFS measured the time from neoadjuvant treatment initiation to the first distant metastasis. The secondary endpoints included overall survival (OS), calculated from the initiation of neoadjuvant treatment to death from any cause.

## Statistical analysis

PSM is a widely used technique for adjusting pre-treatment variables in retrospective studies, effectively mitigating the confounding bias. Propensity scores traditionally predict the likelihood of receiving treatment based on pre-treatment variables. In the case of binary treatment, propensity scores are typically estimated using logistic regression. However, for scenarios involving multiple categorical treatments, such as the three categories in this study, Imbens et al. [18] proposed the generalized propensity scores to account for multiple levels of treatment and suggested that the multinomial logit model, an extension of logistic regression, is more appropriate for estimating propensity scores. In this study, the R statistical software's nnet package was employed to fit multinomial regression models and calculate three generalized propensity scores for each patient. These propensity scores were derived from variables including gender, age, body mass index, tumor distance from the anal verge, clinical T stage, clinical N stage, and mesorectal fascia status. Since each patient possessed three propensity scores which sum to one, only two propensity scores were necessary for matching. To facilitate this, an algorithm developed by Jeremy A. Rassen [19] were used for PSM with three treatments. This algorithm performed ‘within-trio’ optimized matching in a two-dimensional space defined by each patient’s combination of two propensity scores, aiming to identity trios of patients—each receiving one of the three treatments (CRT, STNT, and LTNT)—with the smallest within-trio distance, utilizing a caliper of 0.10. The perimeter of the triangle formed by connecting all trios of patients served as the distance metric in this study.

Before and after PSM, the categorical variables between the three groups were compared using the Chi-square test or Fisher’s exact test, whereas the continuous variables were compared using the Kruskal–Wallis rank-sum test. The survival outcomes were analyzed and compared using the Kaplan–Meier method. All statistical analyses were carried out by R version 4.2.2 (R Foundation for Statistical Computing, Vienna, Austria). A P value < 0.05 was recognized as statistically significant.

## Results

### Patient selection

**Fig. 1:**
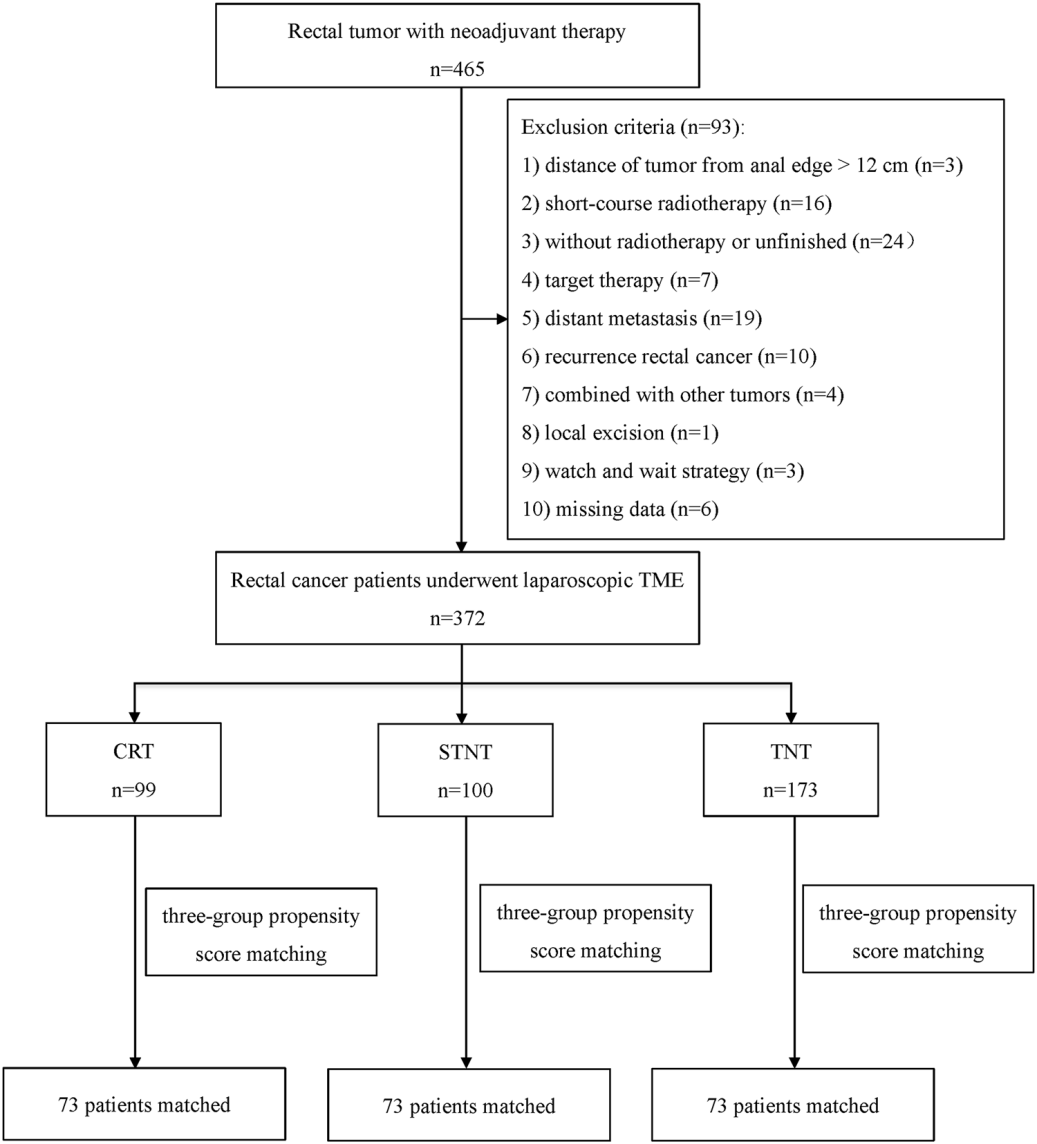
Patient selection diagram. LARC locally advanced rectal cancer, CRT chemoradiotherapy, STNT one to three cycles of chemotherapy with chemoradiotherapy, LTNT four or more cycles of chemotherapy with chemoradiotherapy

From January 2015 to December 2020, a total of 465 patients with rectal cancer underwent neoadjuvant therapy, and 93 patients were excluded according to the predefined exclusion criteria (Fig. 1). Consequently, 372 patients were included in the final analysis, with 99 in CRT group, 100 in STNT group, and 173 in LTNT group. After PSM, each group comprised 73 patients. The data of chemotherapy during TNT was shown in Table S1. CAPOX was administrated for the majority of patients, with sixteen patients receiving FOLFOX and four receiving both.

### Baseline characteristics

Patient characteristics are summarized in Table 1. Before PSM, no significance was identified among the three groups regarding gender, body mass index, the distance of the tumor from the anal verge, clinic T stage, and mesorectal fascia involvement. However, patients in the LTNT group exhibited a lower median age compared to the other two groups (p = 0.010). In addition, the proportion of patients with clinical N positive status was highest in LTNT group, followed by CRT and STNT group (86.6% vs. 81.8% vs. 73.1%, p = 0.023). Following PSM, gender, age, body mass index, the distance of the tumor from the anal verge, clinic T stage, clinic N stage, and mesorectal fascia involvement were all found to be comparable among the three groups.

**Table 1:** Baseline Characteristics

Variable	Before PSM				After PSM			
	CRT N = 99	STNT N = 100	LTNT N = 173	*P*	CRT N = 73	STNT N = 73	LTNT N = 73	*P*
Gender				0.262				0.944
Female	36(36.4%)	36(36.0%)	77(44.5%)		30(41.1%)	28(38.4%)	29(39.7%)	
Male	63(63.6%)	64(64.0%)	96(55.5%)		43(58.9%)	45(61.6%)	44(60.3%)	
Age (years)	59(27–82)	59(28–81)	54(26–80)	0.010	61(31–80)	59(32–76)	56(38–79)	0.557
BMI (kg/m^2^)	22.5(15.9–32.3)	23.4(16.4–31.4)	23.3(16.0–30.4)	0.332	22.6(18.0–32.3)	23.1(16.4–31.4)	23.1(18.2–30.4)	0.836
Distance of the tumor from the anal verge (cm)	5.0(1.0–12.0)	5.0(0–10.0)	5.0(1.0–12.0)	0.992	5.0(1.0–11.0)	5.0(0.5–10.0)	5.0(1.0–12.0)	0.893
Clinic T stage				0.663^*^				0.990
T1-2	0(0)	1(1.0%)	4(2.4%)		0(0)	0(0)	0(0)	
T3	63(64.3%)	64(69.6%)	100(60.2%)		47(64.4%)	50(68.5%)	49(67.1%)	
T4a	23(23.5%)	18(19.6%)	39(23.5%)		19(26.0%)	16(21.9%)	17(23.3%)	
T4b	12(12.2%)	9(9.8%)	23(13.9%)		7(9.6%)	7(9.6%)	7(9.6%)	
Clinic N stage				0.023^*^				0.702
Negative	18(18.2%)	25(26.9%)	22(13.2%)		12(16.4%)	14(19.2%)	16(21.9%)	
Positive	81(81.8%)	68(73.1%)	145(86.8%)		61(83.6%)	59(80.8%)	57(78.1%)	
MRF involvement				0.927^*^				0.981
Negative	57(58.2%)	51(55.4%)	93(56.4%)		43(58.9%)	42(57.5%)	43(58.9%)	
Positive	41(41.8%)	41(44.6%)	72(43.6%)		30(41.1%)	31(42.5%)	30(41.1%)	

Data are number (%) or median (range)

*BMI* body mass index, *MRF* mesorectal fascia, *PSM* propensity score matching, *CRT* chemoradiotherapy, *STNT* one to three cycles of chemotherapy with chemoradiotherapy, *LTNT* four or more cycles of chemotherapy with chemoradiotherapy

^*^Missing values were excluded in the test

### Perioperative and pathological outcomes

Table 2 presents perioperative results. The surgery approach, type of surgery, total postoperative complications, major complications, and postoperative hospital stays did not show significant differences among the three groups. However, prior to PSM, the rates adjuvant chemotherapy progressively decreased across the three study groups, with rates of 66.7% in CRT group, 58.0% in STNT group, and 30.1% in LTNT group (p < 0.001). Even after PSM, the highest rate of adjuvant chemotherapy rate was still observed in CRT group, followed by STNT and LTNT group (71.2% vs. 54.8% vs. 32.9%, p < 0.001).

**Table 2:** Perioperative outcomes

Variable	Before PSM				After PSM			
	CRT N = 99	STNT N = 100	LTNT N = 173	*P*	CRT N = 73	STNT N = 73	LTNT N = 73	*P*
ASA score				0.581				0.897
I-II	80(80.8%)	75(75.0%)	137(79.2%)		58(79.5%)	56(76.7%)	58(79.5%)	
III-IV	19(19.2%)	25(25.0%)	36(20.8%)		15(20.5%)	17(23.3%)	15(20.5%)	
Surgery approach				0.051				0.302
Laparoscopy	78(78.8%)	78(78.0%)	122(70.5%)		60(82.2%)	56(76.7%)	53(72.6%)	
Open	15(15.2%)	21(21.0%)	47(27.2%)		10(13.7%)	16(21.9%)	19(26.0%)	
Conversion	6(6.0%)	1(1.0%)	4(2.3%)		3(4.1%)	1(1.4%)	1(1.4%)	
Type of surgery				0.811				0.291
Low anterior resection	54(54.5%)	60(60.0%)	104(60.1%)		38(52.1%)	47(64.4%)	48(65.7%)	
Intersphincteric resection	15(15.2%)	9(9.0%)	25(14.5%)		13(17.8%)	6(8.2%)	11(15.1%)	
Abdominoperineal resection	27(27.3%)	29(29.0%)	40(23.1%)		19(26.0%)	19(26.0%)	14(19.2%)	
Hartmann	2(2.0%)	2(2.0%)	3(1.7%)		2(2.7%)	1(1.4%)	0(0.0%)	
Transanal total mesorectal excision	1(1.0%)	0(0.0%)	1(0.6%)		1(1.4%)	0(0.0%)	0(0.0%)	
Total postoperative complications	17(17.2%)	24(24.0%)	41(23.7%)	0.393	12(16.4%)	16(21.9%)	16(21.9%)	0.634
Major complications	3(3.0%)	3(3.0%)	6(3.5%)	1.000	1(1.4%)	1(1.4%)	2(2.7%)	1.000
Postoperative hospital stays (days)	7(3–25)	7(3–19)	7(4–35)	0.452	7(3–15)	7(3–19)	7(4–35)	0.306
Adjuvant chemotherapy	66(66.7%)	58(58.0%)	52(30.1%)	< 0.001	52(71.2%)	40(54.8%)	24(32.9%)	< 0.001

Data are number (%) or median (range)

*PSM *propensity score matching, *CRT* chemoradiotherapy, *STNT* one to three cycles of chemotherapy with chemoradiotherapy, *LTNT* four or more cycles of chemotherapy with chemoradiotherapy

Table 3 outlines pathological results. Before PSM, the rate of pCR differed among the three groups, with rates of 10.1% in CRT group, 19.0% in STNT group, and 24.9% in LTNT group (p = 0.012), indicating an ascending trend. After PSM, the pCR rate also increased, but the distinction did not reach statistical significance (11% in CRT, 17.8% in STNT and 26.0% in LTNT, p = 0.062).

**Table 3:** Pathological outcomes

Variable	Before PSM				After PSM			
	CRT N = 99	STNT N = 100	LTNT N = 173	*P*	CRT N = 73	STNT N = 73	LTNT N = 73	*P*
Tumor diameter (cm)	2.3(0–7.0)	2.0(0–11.0)	2.0(0–9.0)	0.106	2.3(0.0–7.0)	2.0(0.0–11.0)	2.0(0.0–7.0)	0.261
CRM				0.814^*^				0.869^*^
Negative	91(95.8%)	89(97.8%)	165(95.9%)		69(98.6%)	65(97.0%)	70(97.2%)	
Positive	4(4.2%)	2(2.2%)	7(4.1%)		1(1.4%)	2(3.0%)	2(2.8%)	
ypT stage				0.046				0.075
Tis	2(2.0%)	1(1.0%)	0(0.0%)		1(1.4%)	1(1.4%)	0(0.0%)	
T0	11(11.1%)	19(19.0%)	46(26.6%)		9(12.3%)	13(17.8%)	22(30.1%)	
T1	4(4.0%)	6(6.0%)	7(4.0%)		2(2.7%)	4(5.5%)	3(4.1%)	
T2	24(24.3%)	30(30.0%)	43(24.9%)		15(20.6%)	24(32.9%)	16(21.9%)	
T3	55(55.6%)	39(39.0%)	68(39.3%)		43(58.9%)	28(38.3%)	27(37.0%)	
T4	3(3.0%)	5(5.0%)	9(5.2%)		3(4.1%)	3(4.1%)	5(6.9%)	
ypN stage				0.237				0.223
N0	74(74.7%)	73(73.0%)	130(75.2%)		52(71.2%)	54(74.0%)	53(72.6%)	
N1	19(19.2%)	23(23.0%)	26(15.0%)		15(20.6%)	17(23.3%)	11(15.1%)	
N2	6(6.1%)	4(4.0%)	17(9.8%)		6(8.2%)	2(2.7%)	9(12.3%)	
pCR	10(10.1%)	19(19.0%)	43(24.9%)	0.012	8(11.0%)	13(17.8%)	19(26.0%)	0.062
Tumor regression grade				0.143^*^				0.169^*^
Grade 0–1	31(32.6%)	34(36.5%)	81(46.8%)		21(29.6%)	25(36.8%)	35(47.9%)	
Grade 2	54(56.9%)	50(53.8%)	73(42.2%)		43(60.5%)	36(52.9%)	35(48.0%)	
Grade 3	10(10.5%)	9(9.7%)	19(11.0%)		7(9.9%)	7(10.3%)	3(4.1%)	
Lymphovascular invasion	9(9.1%)	10(10.0%)	11(6.4%)	0.516	8(11.0%)	7(9.6%)	3(4.1%)	0.281
Perineural invasion	23(23.2%)	16(16.0%)	36(20.8%)	0.427	18(24.7%)	13(17.8%)	12(16.4%)	0.408
MMR status				0.137^*^				0.520^*^
proficient MMR	68(95.8%)	66(100%)	99(94.3%)		51(96.2%)	49(100%)	43(97.7%)	
deficient MMR	3(4.2%)	0(0%)	6(5.7%)		2(3.8%)	0(0%)	1(2.3%)	
ypT downstaging				0.642^*^				0.368
Yes	63(64.3%)	62(67.4%)	116(69.9%)		44(60.3%)	52(71.2%)	49(67.1%)	
No	35(35.7%)	30(32.6%)	50(30.1%)		29(39.7%)	21(28.8%)	24(32.9%)	
ypN downstaging				0.923^*^				0.635
Yes	57(70.4%)	47(69.1%)	104(71.7%)		41(67.2%)	43(72.9%)	37(64.9%)	
No	24(29.6%)	21(30.9%)	41(28.3%)		20(32.8%)	16(27.1%)	20(35.1%)	

Data are number (%) or median (range)

*CRM* circumferential resection margin,* pCR* pathological complete response, *PSM* propensity score matching, *CRT* chemoradiotherapy, *STNT* one to three cycles of chemotherapy with chemoradiotherapy, *LTNT* four or more cycles of chemotherapy with chemoradiotherapy, *MMR* mismatch repair

^*^Missing values were excluded in the test

### Survival outcomes

**Fig. 2:**
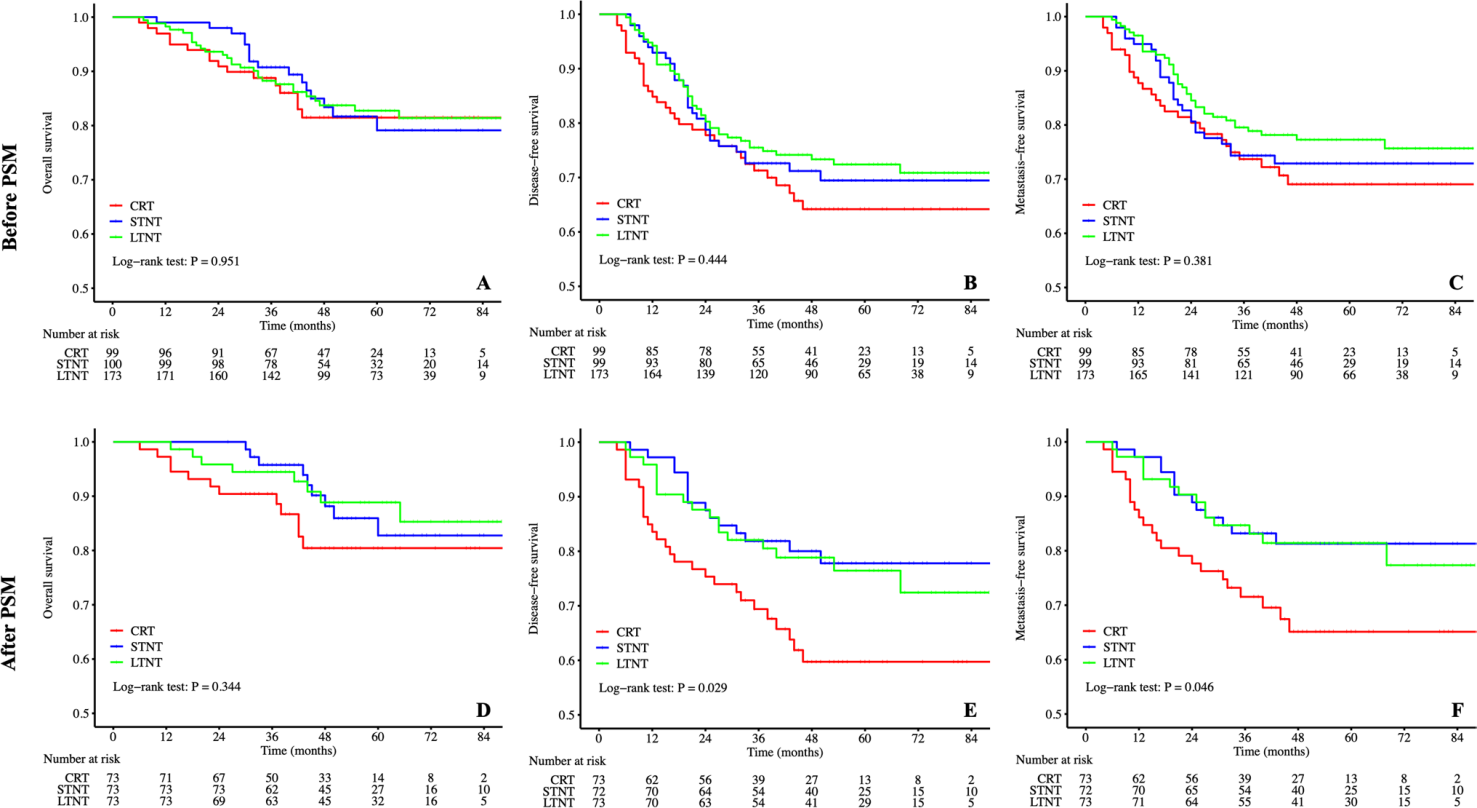
Kaplan-Meier curves of overall survival, disease-free survival, and metastasis-free survival. PSM propensity score matching, CRT chemoradiotherapy, STNT one to three cycles of chemotherapy with chemoradiotherapy, LTNT four or more cycles of chemotherapy with chemoradiotherapy

The median follow-up duration was 50 months (range, 6–111 months). Sixty patients in total passed away during the study period (16 in CRT, 16 in STNT, and 28 in LTNT). Additionally, 11 patients experienced local recurrence (1 in CRT, 2 in STNT, and 8 in LTNT), while 92 patients developed distant metastasis (28 in CRT, 26 in STNT, and 38 in LTNT) (Table 4). Kaplan–Meier survival curves for OS, DFS, and MFS are presented in Fig. 2.

**Table 4:** Survival outcomes

Variable	Before PSM				After PSM			
	CRT N = 99	STNT N = 100	LTNT N = 173	*P*	CRT N = 73	STNT N = 73	LTNT N = 73	*P*
Total death	16(16.2%)	16(16.0%)	28(16.2%)	1.000	12(16.4%)	9(12.3%)	8(11.0%)	0.596
Total local recurrence	1(1.0%)	2(2.0%)	8(4.6%)	0.211	1(1.4%)	1(1.4%)	3(4.1%)	0.623
Total distant metastasis	28(28.3%)	26(26.3%)	38(22.0%)	0.472	23(31.5%)	13(18.1%)	14(19.2%)	0.101
3-year OS rate	88.8%	90.7%	88.3%	0.825	90.4%	95.8%	94.5%	0.476
3-year DFS rate	71.3%	72.6%	75.5%	0.725	68.8%	81.9%	82.1%	0.088
3-year MFS rate	73.7%	74.4%	79.5%	0.462	71.5%	83.2%	84.7%	0.094
5-year OS rate	81.5%	79.1%	82.7%	0.762	80.4%	82.8%	88.8%	0.362
5-year DFS rate	64.2%	69.5%	72.4%	0.369	59.7%	77.8%	76.5%	0.027
5-year MFS rate	69.0%	72.9%	77.3%	0.313	65.1%	81.3%	81.4%	0.030

*OS* overall survival, *DFS* disease-free survival, *MFS* metastasis-free survival, *PSM* propensity score matching, *CRT* chemoradiotherapy, *STNT* one to three cycles of chemotherapy with chemoradiotherapy, *LTNT* four or more cycles of chemotherapy with chemoradiotherapy

Prior to PSM, there were no significant difference observed among the three groups in terms of OS (p = 0.951, Fig. 2A), DFS (p = 0.444, Fig. 2B), or MFS (p = 0.381, Fig. 2C). Following PSM, OS remained comparable among the three groups (p = 0.344, Fig. 2D). However, DFS differed significantly between the three groups (p = 0.029, Fig. 2E), attributed to a lower 5-year DFS rate in CRT group compared with STNT and LTNT groups (59.7% vs. 77.8% vs. 76.5%, p = 0.027, Table 4). Similarly, MFS exhibited a significant difference among the three groups (p = 0.046, Fig. 2F), driven by a lower 5-year MFS rate in CRT group than in STNT and LTNT groups (65.1% vs. 81.3% vs. 81.4%, p = 0.030; Table 4). Notably, there were no significant differences in DFS or MFS between STNT and LTNT arms.

### Subgroup analysis

**Fig. 3:**
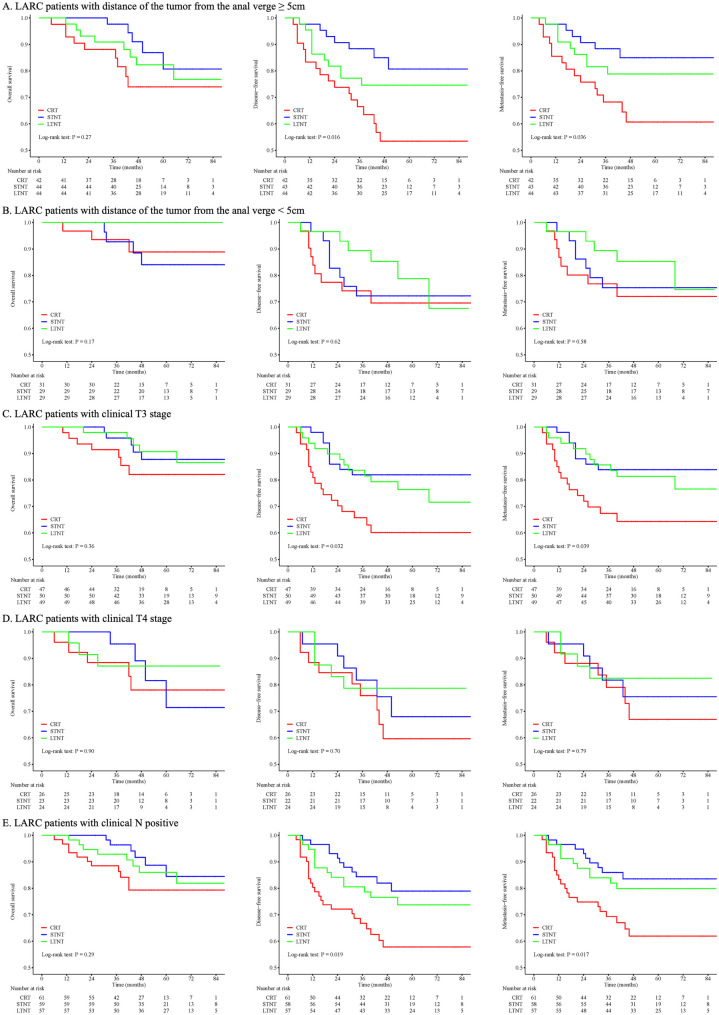

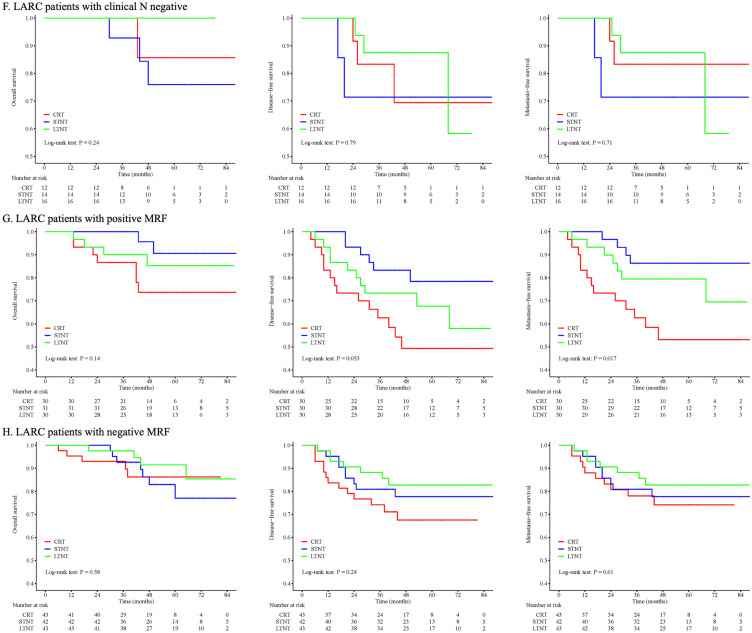
Kaplan-Meier curves of overall survival, disease-free survival, and metastasis-free survival for subgroup analysis. LARC locally advanced rectal cancer, MRF mesorectal fascia, CRT chemoradiotherapy, STNT one to three cycles of chemotherapy with chemoradiotherapy, LTNT four or more cycles of chemotherapy with chemoradiotherapy

Following PSM, we conducted subgroup analyses to investigate survival outcomes regarding specific clinical characteristics, including the distance of the tumor from the anal verge, clinical T stage, clinical N stage, and mesorectal fascia involvement (Fig. 3).

Among the cohort of 130 patients with distance of the tumor from the anal verge ≥ 5 centimeters, no statistically significant difference was observed in OS among the three groups (p = 0.27). However, there were notable distinctions in DFS (p = 0.016) and MFS (p = 0.036) among these groups. No discernible disparity in DFS or MFS was identified between the STNT and LTNT arms (Fig. 3A). In contrast, among the 89 patients with the distance of the tumor from the anal verge < 5 centimeters, no significant difference in OS (p = 0.17), DFS (p = 0.62), and MFS (p = 0.58) was observed across the three groups (Fig. 3B).

For the 146 patients diagnosed with clinical T3 stage tumors, OS remained similar (p = 0.36), whereas DFS (p = 0.032) and MFS (p = 0.039) exhibited substantial discrepancies among the three groups. There was no distinction in DFS or MFS between the STNT and LTNT arms (Fig. 3C). Simultaneously, for the 73 patients diagnosed with clinical T4 stage tumors, there were marginal variations in OS (p = 0.90), DFS (p = 0.70), and MFS (p = 0.79) among the three groups (Fig. 3D).

Among the 177 patients diagnosed with clinical N positive tumors, OS did not show a significant difference (p = 0.29), while DFS (p = 0.019) and MFS (p = 0.017) displayed notable distinctions across the three groups. No significant differences in DFS or MFS were observed between the STNT and LTNT arms (Fig. 3E). On the contrary, in the cohort of 42 patients diagnosed with clinical N negative tumors, OS (p = 0.24), DFS (p = 0.79), and MFS (p = 0.71) were all comparable among the three groups (Fig. 3F).

For the 91 patients diagnosed with clinical positive mesorectal fascia involvement, the analysis revealed no significant differences in OS (p = 0.14) and DFS (p = 0.053), while MFS (p = 0.017) differed significantly among the three groups. Similar to previous analyses, there was no distinction in MFS between the STNT and LTNT arms (Fig. 3G). In addition, in the cohort of 128 patients diagnosed with clinical negative mesorectal fascia involvement, no statistically significant differences were observed in the OS (p = 0.58), DFS (p = 0.24), and MFS (p = 0.61) among these groups (Fig. 3H).

As for tumor down-staging, there were 145 patients with T downstaging and 74 without T downstaging after neoadjuvant therapy. Although subgroup analyses showed a better DFS and MFS for patients with STNT and LTNT arms, it failed to gain a statistical significance (Fig. S1A and B). There were 121 patients with N downstaging after neoadjuvant therapy, both STNT and LTNT arms exhibited significantly better DFS (p = 0.002) and MFS (p =0.001) compared with CRT arms, with no distinction in DFS or MFS between the STNT and LTNT arm. There was no significant difference in DFS and MFS among three groups for patients without N down-staging (Fig. S1C and D). OS was comparable among three arms regardless of T or N downstaging.

## Discussion

TNT has emerged as a promising treatment approach for LARC. Nevertheless, the optimal cycles of chemotherapy during TNT remainunknown. In this study, we employed PSM analysis to compare the prognostic efficacy of STNT, LTNT, and CRT for patients with LARC. Our findings indicated that both STNT and LTNT conferred a significant advantage in terms of improving DFS and MFS compared to CRT, albeit without a notable impact on OS. Notably, this survival benefit was maintained in the subgroup analyses, encompassing patients with a tumor distance from the anal verge ≥ 5 cm, clinical T3 stage, clinical N positive status, and positive mesorectal fascia involvement.

One of the primary objectives for implementing TNT is to improve the prognosis of LARC patients. Various studies have previously compared the survival outcomes of TNT with those of CRT. For instance, the Spanish GCR-3 phase II trial reported similar 5-year DFS rates between TNT with four cycles of CAPOX and CRT (62% vs. 64%) after a median follow-up of 69 months [20]. Another large cohort study from MSKCC likewise found no significant difference in DFS and MFS between TNT with 4 months of chemotherapy and CRT [8]. However, it is crucial to acknowledge the significant disparities in baseline characteristics between these two studies. The TNT group enrolled more patients with T4 lesions and grade 3 tumors in Spanish GCR-3 trial and more patients with T4 lesions and clinical N positive status in MSKCC cohort study, which could have an impact on the survival results [8, 20]. In our current study, prior to PSM, notable differences in age and clinical N positive status existed among the three treatment groups. Similarly, no significant differences were found among the three groups regarding DFS or MFS. Nevertheless, following PSM, these baseline discrepancies among groups were mitigated and the results revealed improved DFS and MFS in both STNT and LTNT compared to CRT, with no difference between STNT and LTNT. This result was supported by the TIMING trial, which demonstrated that patients receiving TNT with two, four, or six cycles of chemotherapy exhibited superior DFS compared to CRT. Intriguingly, our study, like the Timing trial, found that the number of chemotherapy cycles during TNT did not exert a significant effect on survival outcomes [12], a phenomenon warranting further investigation. In terms of OS, our study, consistent with recent studies, including the PRODIGE-23 trial [6, 8, 21], failed to demonstrate a notable advantage for TNT. It is crucial to recognize that OS can be affected by various factors beyond tumor recurrence and metastasis, including comorbidities and accident, making it challenging to attribute the impact solely to TNT. Therefore, the potential beneficial of TNT in improving prognosis cannot be denied, given the similarity in OS outcomes.

To further identity suitable patient populations for TNT, we conducted subgroup analyses according to four key characteristics. These analyses revealed that the survival advantage of TNT persisted among patients with specific characteristics, including tumor distance from the anal verge ≥ 5 cm, clinical T3 stage, clinical N positive status, or positive mesorectal fascia involvement. Like our study, Zhang et al. [22] also demonstrated that for patients with positive mesorectal fascia involvement or clinical N2, TNT decreased the 3-year distant metastasis rate. While no significant differences were observed between TNT and CRT in other subgroup analyses, it is essential to acknowledge the limitations imposed by small sample sizes. Consequently, additional studies with larger cohorts are warranted for further investigation.

The pCR rates serve as a pivotal indicator of the neoadjuvant treatment efficacy. Previous literature has reported pCR rates ranging from 5.8% to 21.8% in CRT, 13.6% to 22.7% in STNT, and 27.1% to 38.5% in LTNT, respectively [6, 10, 11, 23, 24]. Our current study was consistent with these reported figures. In addition, our previous work demonstrated that compared with CRT, both TNT with one to two cycles or ≥ 3 cycles of chemotherapy increased the pCR rate [9], which was further supported by the TIMING trial, indicating that increased chemotherapy cycles during TNT aligned with higher pCR rates [11]. Consistent with these studies, the pCR rates still exhibited an ascending trend after PSM in the current research. However, it failed to reach a statistical difference, possibly attributed to limitations arising from the relatively small sample size, which restricted the statistical power to detect differences among the groups.

The strength of the present study was that we employed PSM analysis to mitigate confounding bias among the three groups, and with a long-term follow-up period, our investigation revealed that both STNT and LTNT were associated with improvements in DFS and MFS compared with CRT. In addition, our subgroup analyses unveiled that LARC patients with one of four tumor characteristics mentioned above may experience notable benefits from TNT. However, some limitations in our study needed to be acknowledged. Firstly, this was a single-center study with a relatively modest sample size. Secondly, although all data were collected from our prospective database, the analysis was conducted retrospectively. Thirdly, some important parameters, including extramural vascular invasion (EMVI), clinical response to neoadjuvant therapy, as well as chemotherapy-induced toxicities were not reported. Fourthly, patients with watch and wait strategy were excluded because this strategy was not routinely carried out in our hospital during this period. Although the phase II Organ Preservation in Rectal Adenocarcinoma (OPRA) trial demonstrated that organ preservation was achievable in about half of patients treated with TNT with impressive prognostic outcomes [25], more studies with longer follow-up and larger sample sizes are still needed. Fifthly, we excluded patients receiving short-course radiotherapy. The RAPIDO trial observed a significant decline in 3-year cumulative distant metastasis rate from a TNT approach (short-course radiotherapy with consolidation chemotherapy) [26]. However, the TNT group showed significantly higher locoregional recurrence rates compared to CRT group after 5-year follow-up, suggesting the necessity for further refinement of TNT with short-course radiotherapy [27]. Furthermore, while we reported the rate of adjuvant chemotherapy, we did not calculate the total number of chemotherapy cycles (combining neoadjuvant and adjuvant phases). As a consequence, it remains uncertain whether the observed differences in DFS and MFS among the three groups were primarily attributed to variations in the cycles of chemotherapy during TNT or total cycles of chemotherapy encompassing both neoadjuvant and adjuvant phases. There was one study reported that, despite the similar total cycles of chemotherapy, TNT led to improved DFS, but the sample size was relatively small [12], warranting further evidence. Finally, the timing of chemotherapy administration (induction and/or consolidation) may exert an impact on survival outcomes. Nevertheless, we did not undertake this subgroup analysis due to the small sample size. So far, only a limited number of studies have simultaneously assessed the impact of the timing and duration of chemotherapy during TNT on survival outcomes. Future investigations, such as individual patient data meta-analyses derived from randomized trials and network meta-analyses, may provide insights into this complicated question. Regardless of these limitations, we believe that our study offers valuable insights into the treatment of LARC.

Overall, our study underlines that compared with CRT, both STNT and LTNT can improve the DFS and MFS in the treatment of LARC. Importantly, these survival outcomes are indistinguishable between STNT and LTNT. Furthermore, our findings suggest that LARC patients with specific clinical characteristics, including the distance of the tumor from the anal verge ≥ 5 cm, clinical T3 stage, clinical N positive status, or positive mesorectal fascia involvement, may particularly benefit from TNT. We expect that the results of ongoing randomized trials (NCT02843191, NCT03177382, and NCT04747951) will corroborate the prognostic value of TNT in LARC treatment.

## Conclusion

Both STNT and LTNT demonstrated improved DFS and MFS outcomes, which suggests that TNT should be preferable neoadjuvant treatment for LARC compare to CRT, especially for patients with tumor distance from the anal verge ≥ 5 cm, clinical T3 stage, clinic positive N stage, or involved mesorectal fascia. Notably, survival outcomes were similar between STNT and LTNT, suggesting that chemotherapy cycles in TNT may not significantly impact survival.

## Electronic supplementary material

Below is the link to the electronic supplementary material.

Supplementary Material 1 (DOCX 356 KB)

## Data Availability

Summarized data will be made available on request.

## Abbreviations

TNT: Total neoadjuvant therapy
LARC: Locally advanced rectal cancer
TME: Total mesorectal excision
CRT: Long-course chemoradiotherapy
STNT: Long-course chemoradiotherapy with one to three cycles of chemotherapy
LTNT: Long-course chemoradiotherapy with four or more cycles of chemotherapy
PSM: Propensity score matching
DFS: Disease-free survival
MFS: Metastasis-free survival
OS: Overall survival
pCR: Pathological complete response
CAPOX: Oxaliplatin and capecitabine
FOLFOX: Oxaliplatin, leucovorin, and fluorouracil
