# Supplementary material for: Total neoadjuvant therapy for locally advanced rectal cancer: a three-group propensity score matched study
Source: Int J Colorectal Dis. 2024 Mar 16;39(1):38. doi: 10.1007/s00384-024-04610-1 (PMC10944449; doi:10.1007/s00384-024-04610-1)
Supplement: Supplementary file 1 — Supplementary Material 1 (DOCX 356 KB) [file 384_2024_4610_MOESM1_ESM.docx]

**Supplementary materials**

Table S1. Details of systemic chemotherapy in total neoadjuvant therapy.

| Characteristics | Before PSM | | | After PSM | | |
| --- | --- | --- | --- | --- | --- | --- |
|  | CRT  N = 99 | STNT  N = 100 | LTNT  N = 173 | CRT  N = 73 | STNT  N = 73 | LTNT  N = 73 |
| Systemic chemotherapy |  |  |  |  |  |  |
| Received CAPOX | - | 85(85%) | 155(89.6%) | - | 67(91.8%) | 65(89.0%) |
| Received FOLFOX | - | 7(7.0%) | 9(5.2%) | - | 3(4.1%) | 2(2.7%) |
| Received CAPOX and FOLFOX | - | 0(0%) | 4(2.3%) | - | 0(0%) | 3(4.1%) |
| Unknown | - | 8(8.0%) | 5(2.9%) | - | 3(4.1%) | 3(4.1%) |
| Cycles of systemic chemotherapy | - | 2(1, 3) | 6(5, 6) | - | 2(1, 3) | 6(5, 6) |

Data are number (%) or median (IQR), PSM propensity score matching, CRT chemoradiotherapy, STNT one to three cycles of chemotherapy with chemoradiotherapy, LTNT four or more cycles of chemotherapy with chemoradiotherapy, IQR interquartile range.


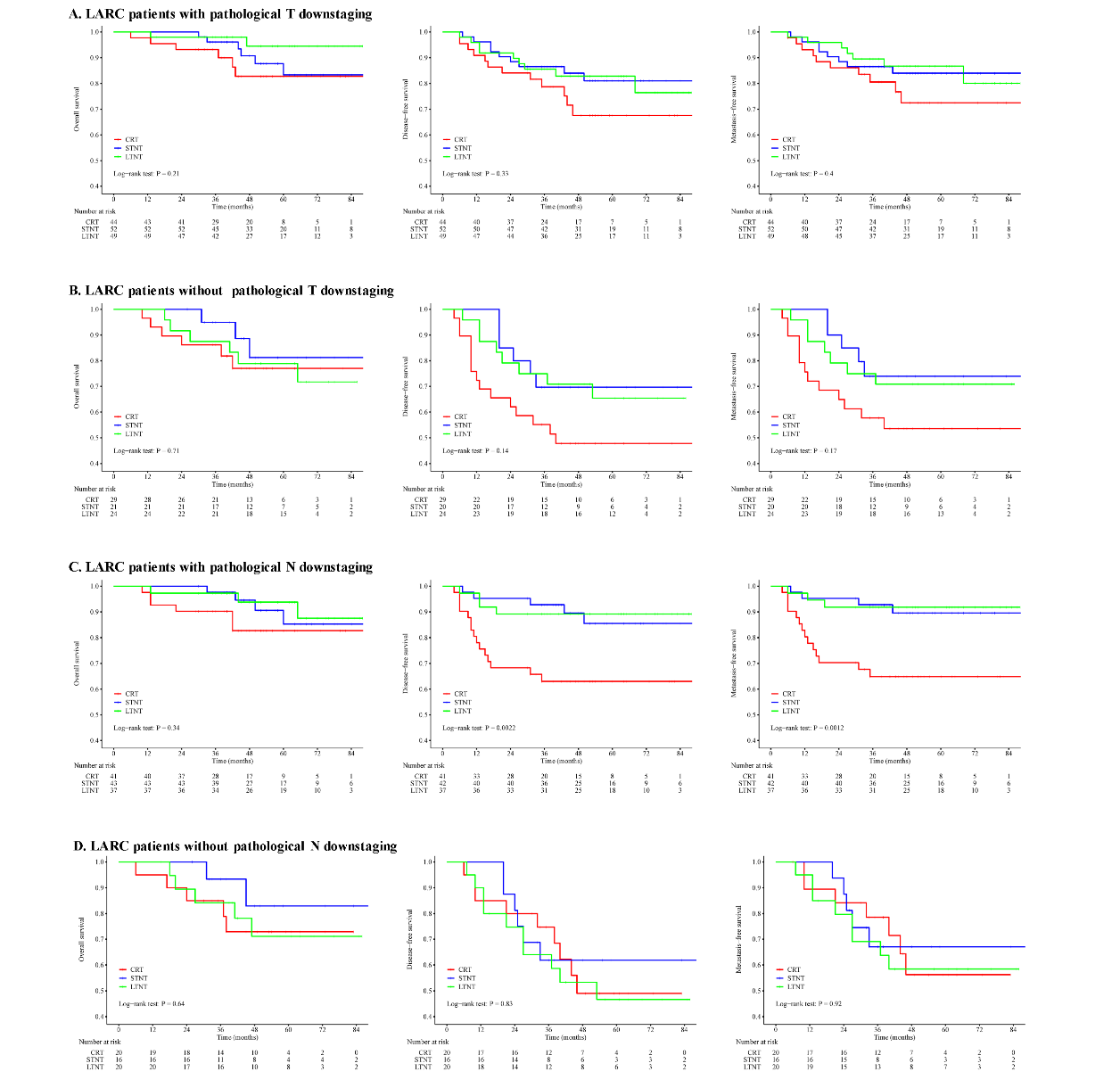


Figure S1. Kaplan-Meier curve of overall survival, disease-free survival, and metastasis-free survival for subgroup analysis. LARC locally advanced rectal cancer, CRT chemoradiotherapy, STNT one to three cycles of chemotherapy with chemoradiotherapy, LTNT four or more cycles of chemotherapy with chemoradiotherapy.
